# Durability Testing of a Polymer Worm Gear Used in a Vehicle Steering System

**DOI:** 10.3390/ma18184236

**Published:** 2025-09-09

**Authors:** Jakub Franiasz, Tomasz Machniewicz

**Affiliations:** 1 AGH University of Krakow, Faculty of Mechanical Engineering and Robotics, al. Mickiewicza 30, 30-059 Kraków, Poland; 2 Nexteer Automotive Poland Sp. z o. o., ul. Towarowa 6, 43-100 Tychy, Poland

**Keywords:** durability, vehicle electric power steering system, worm gear, steer by wire, polymer gear

## Abstract

Polymer worm gears are increasingly utilized in electric power steering (EPS) systems due to their favorable manufacturing features and performance. Ensuring consistent mechanical properties under various operating conditions is critical for steering reliability throughout a vehicle’s lifespan. This study investigates the durability of injection-molded polyamide 66 worm gears within a Pinion-EPS configuration, where torque from the assist motor is transmitted through a worm–worm gear set to the rack and ultimately to the vehicle wheels. Given the complexity of steering maneuvers and the absence of mechanical integrity in steer-by-wire systems, durability testing becomes essential to understand if the considered worm gear for a certain steering system application provides safety and the needed performance within a specified product service life. This paper compares multiple testing methodologies. Traditional approaches, such as maximum torque and rotational speed, prove insufficient for comprehensive durability assessment, especially considering the thermal sensitivity of polymer materials. The findings highlight the limitations of conventional testing methods and emphasize the need for application-specific testing methods that reflect real-world boundary conditions. This research contributes to the development of more accurate and reliable evaluation techniques for polymer gear components in modern EPS systems, with implications for both conventional and autonomous vehicle platforms.

## 1. Introduction

Polymer gears are widely used in steering systems on the automotive market. There are several reasons for this, like ease of manufacturing, relatively low cost, compact size, excellent NVH performance, and decent mechanical properties. In particular, these are variables expected to remain unchanged or within the needed range for all of the working conditions that the steering system is exposed to. Considering the complexity of steering maneuvers being performed during vehicle usage, the plastic worm gear works at various loads. In this paper, the problem of durability testing is described, which is crucial to understanding the reliability of the steering system, which must be ensured throughout the vehicle’s entire defined life [1,2,3,4].

The EPS (electric power steering) system is an inherent part of most passenger vehicles on the modern automotive market. EPS allows for a reduction in the driver’s effort during car operation, keeping the steering feel pleasant during any driving condition on various surfaces. Also, there are legal regulations that require several safety functions to be provided by the EPS system, e.g., lane departure warning or ESP system support. Moreover, safety is extremely important for steer-by-wire EPS systems. Its significance is related to lack of mechanical connection between the handwheel and steering gear—any excessive resistance on the steering kinematic path cannot be compensated by driver effort. Indeed, potential steer-by-wire issues are a matter of autonomous vehicle applications as well [2,5,6].

**Figure 1:**
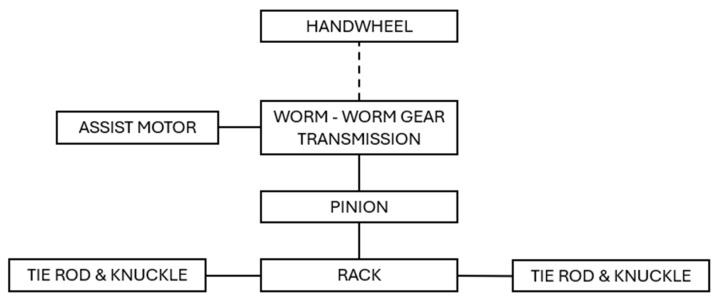
Pinion-EPS system schematic.

Basically, the EPS system consists of the assist motor (equipped with a controller) that provides torque to further steer gear components. In this paper, a Pinion-EPS application is considered, where assist motor torque is transferred to worm–worm gear transmission, which multiplies the torque and applies it to the pinion. This transfers the torque to the rack, which moves linearly, to steer the tie rods and knuckles connected to the vehicle wheels (shown in Figure 1). The entire kinematic path must be accurate, precise, quietly operable, and provide a proper feeling with no unintended events during handwheel turning. All of the mentioned features must remain within an acceptable range for the entire specified lifetime that the vehicle is designed for. Therefore, the durability aspect is key as mechanical properties are strongly related to components’ behavior, determining comfortable usage [7,8,9,10,11].

The typical durability definition for the EPS system is a sequence of handwheel maneuvers under specific loads repeated a certain number of times. Of course, vehicle usage is not strictly predictable, so the durability profile may seem unreal in terms of its severity. However, such an approach is reasonable from a safety standpoint, whereby the EPS steering system must be fully functional even at boundary working conditions—assuming the car’s max allowable gross weight, a vehicle positioned on a ramp with the steering axle directed downwards, and reduced tire air pressure. In this paper, the worm gear durability testing method is considered to be appropriately selected for a certain vehicle application. There is no versatile way to assess worm gear durability based on its maximum output torque and rotational speed, which are typical parameters found in the literature [12,13,14,15,16].

The results of specific testing methods might not be conclusive for applications working under different conditions. Since the worm gear is made of polymer, the thermal aspect becomes significant, perhaps equally important as the carried load [17,18,19,20,21,22].

**Figure 2:**
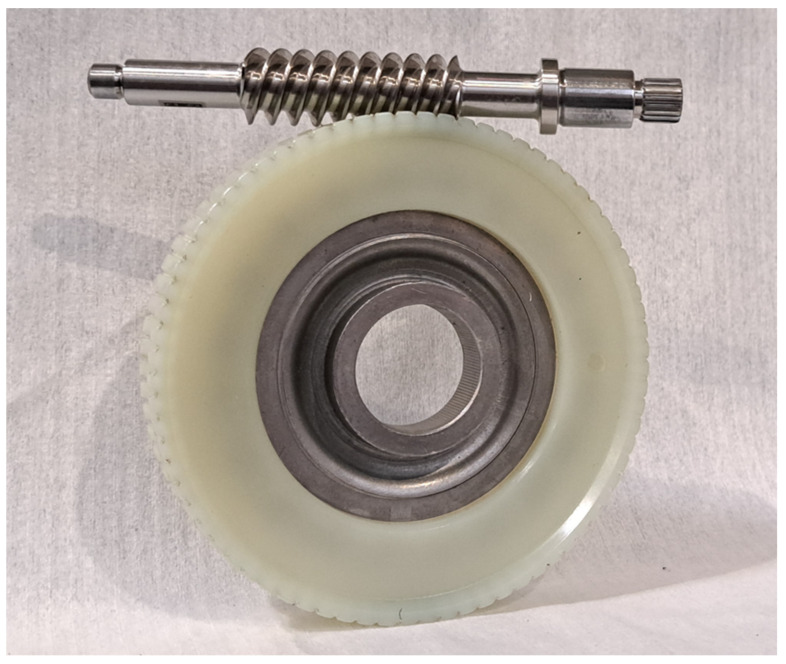
Worm–worm gear transmission used in the EPS system.

In this study, different methods of worm gear durability testing are compared to assess their applicability for vehicle EPS steering system applications. For all testing methods, the same design of worm–worm gear set was used. Since testing leads to component destruction, each test had to be conducted on a separate sample. The worm gear was produced via injection molding using polyamide 66 (all samples come from the same production batch). During sample testing, the remaining samples required proper storage; worm gears were sealed in foil with a humidity absorber. The low number of samples is related to the testing duration. It is a time-consuming process for some cases reviewed in this article. This study was carried out to find a proper method for further testing. Worm–worm gear transmission is shown in Figure 2.

## 2. Materials and Methods

**Figure 3:**
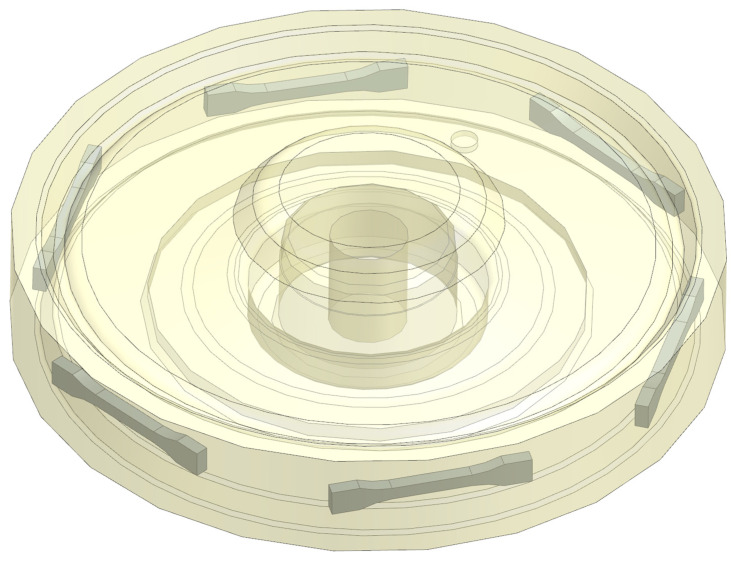
Areas and orientation of specimens machined from molded worm gears.

**Figure 4:**
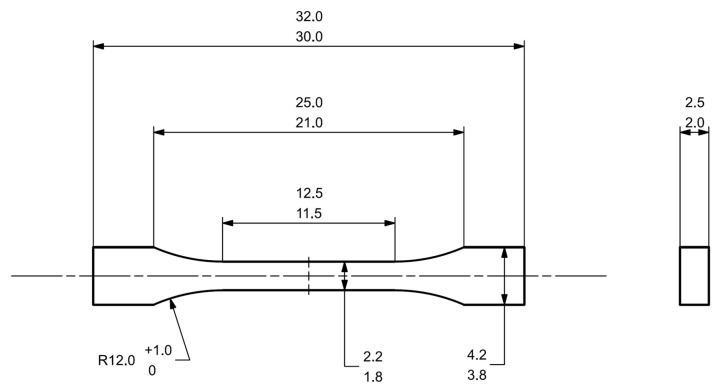
Tensile strength specimen geometry 1 BB per ISO 527.

The main problem considered in this paper is the durability of the worm gear used in electric power steering systems. The worm gear is made of polyamide 66 and works with a steel worm. The basic material and geometrical properties are presented in Table 1. Due to the durability nature of polymers, the polyamide worm gear becomes a key node in the entire kinematic chain, which determines the functionality of the entire steering system [23]. Because of the aspects mentioned above, there is a need to analyze the material properties of the polymer used to manufacture the worm gear. To define basic material properties, the tensile specimens were prepared from previously molded worm gears using the standard manufacturing process. Areas and orientation of specimens machined from worm gears are presented in Figure 3. Because of the relatively small gear size, the smallest specimen size specified by standard ISO 527 was chosen. The specimen geometry is shown in Figure 4.

**Figure 5:**
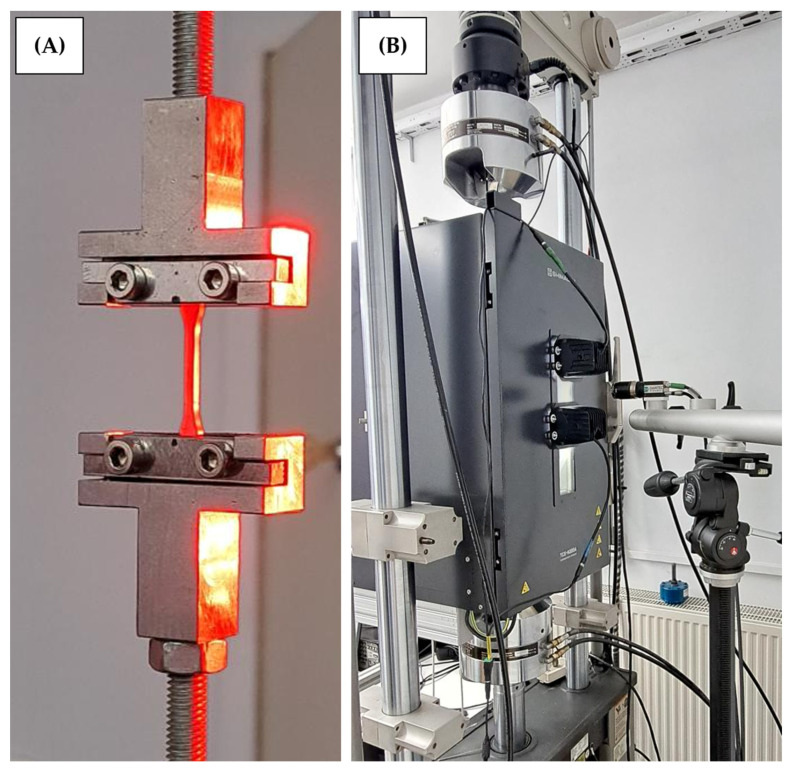
Tensile testing: (**A**) 1 BB specimen installed in machine jaws and (**B**) thermal chamber set up during the test performed.

Tensile strength testing was performed on a hydraulic tensile machine, MTS 810 (manufactured by MTS systems, Eden Prairie, MN 55344, USA), using the DIC method. In the steering system, the worm working with the worm gear generates heat due to friction that results in polyamide strength degradation [24,25,26]. Tensile strength testing was performed at three ambient temperatures: 25, 50, and 75 °C. To provide appropriate thermal conditions during testing, a Shimadzu thermal chamber was used. The test stand is shown in Figure 5.

**Figure 6:**
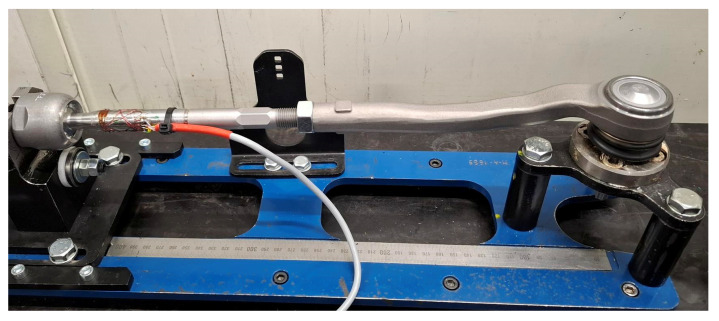
Strain gage installed on the steering system’s inner tie rod.

**Figure 7:**
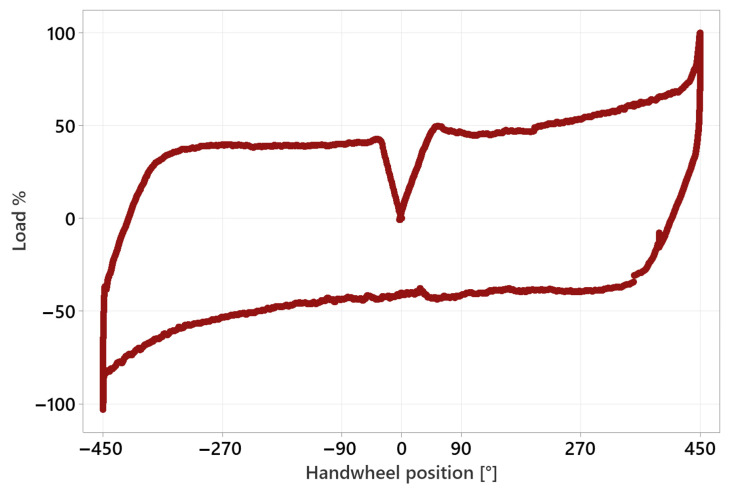
Record of parking maneuver course.

Ambient temperature values for tensile strength testing were set based on worm gear durability testing using a dedicated test stand. To define worm gear durability, it is important to apply a specific sequence of maneuvers at a certain load and number of repetitions, which reflect vehicle utilization within an assumed time period [27]. To reproduce the vehicle’s actual operating conditions in the worm gear, the test profile was created based on data recorded during vehicle operation in the parking area, where steering system loading also depends on vehicle suspension performance [28]. To gather the data, strain gages were applied to the vehicle steering system’s inner tie rods (Figure 6). The vehicle was loaded to its maximum permissible gross weight and placed on a dry, concrete surface. The maneuver was performed from the center to the end of the steering wheel course, then to the opposite end of the steering wheel course, and then returning to the starting position. The steering wheel rotational speed was maintained at approximately 30 RPM. The record is shown in Figure 7.

**Figure 8:**
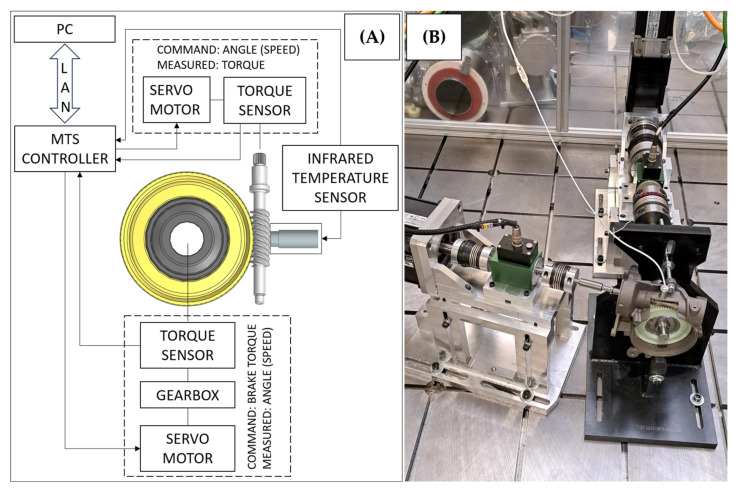
Worm–worm gear test stand: (**A**) schematic and (**B**) actual photo of the stand. Arrows indicate devices signal transfer direction. Dotted boxes group input and output sections.

A dedicated test stand was used to test the worm gear’s durability. This stand allows the worm to be driven and the worm gear to be loaded with a specified torque. Two sets of servomotors, torque sensors, and couplings were used for this purpose. The tested components were mounted in dedicated fixtures. The test stand was equipped with a contactless infrared temperature sensor, which measures the worm temperature during testing. Figure 8 shows a schematic and actual photo of the stand.

The test stand is operated by the MTS FlexTest controller (manufactured by MTS sys-tems, Eden Prairie, MN 55344, USA) and MTS Multipurpose Testware software, version 793. The controller and software fully control the drives and the appropriate loading of the tested system. They also allow us to define test termination conditions, for example, by detecting excessive resistance in the worm gearing system caused by tooth damage. Tests are conducted until the worm gear is destroyed, as this result clearly determines the loss of the ability to transfer assist torque from the motor to the rest of the steering system components.

**Figure 9:**
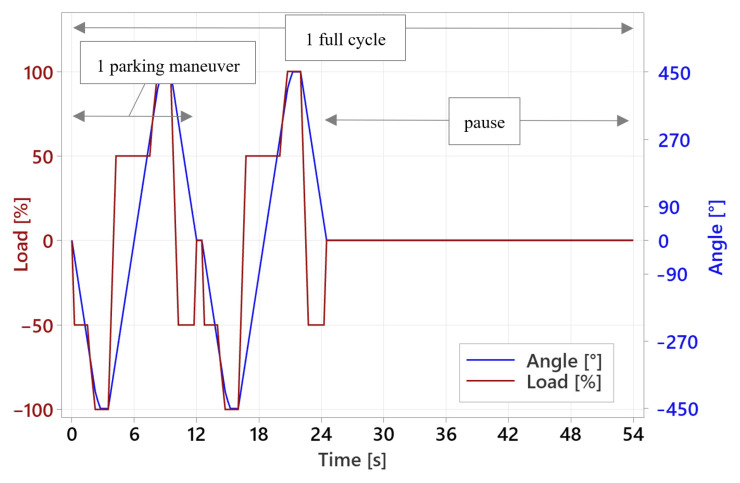
Parking sequence on a timescale.

**Figure 10:**
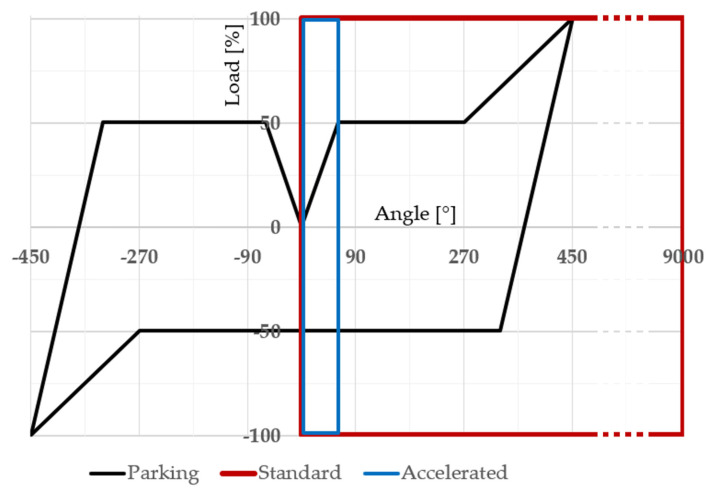
Test profiles comparison.

The nominal peak load of the tested worm gear was set to 110 Nm. One complete parking profile test sequence consists of two consecutive parking maneuvers and a 30 s break. The time course of the test sequence is shown in Figure 9. The results of these tests accurately reflect durability in terms of the number of cycles performed, but their nature makes the testing time-consuming and does not allow for the collection of a sufficient number of results in a reasonable time (one test takes approximately three weeks). Hence, there was a need to define a different test profile that would allow the data of worm gear durability to be obtained in a shorter timeframe. Following the literature [22,29,30,31], a test was developed that uniformly loads the entire circumference of the worm gear by operating under constant conditions. The test profile, titled ‘standard’, consists of 25 clockwise and 25 counterclockwise rotations. The worm gear speed is 30 RPM. The test was conducted with a worm gear load of 110 Nm and 60 Nm. In an attempt to find an appropriate testing method that allows reliable results to be obtained in a relatively short time, an ‘accelerated durability test’ was developed, which is limited to full loading of the section of the worm gear circumference that meshes with the worm during loading maneuver and assumes the engagement and disengagement of all worm gear teeth in contact with the worm. As in previous tests, the rotational speed was set to 30 RPM, and the test was conducted with a nominal worm gear load of 110 Nm. A single test took up to 4 days. All described test profiles are shown in Figure 10. The load is presented on a percentage scale due to different load values assumed for individual samples. The angular course of the worm gear is expressed in degrees because the course remained constant for a given profile regardless of the load.

## 3. Results

**Figure 11:**
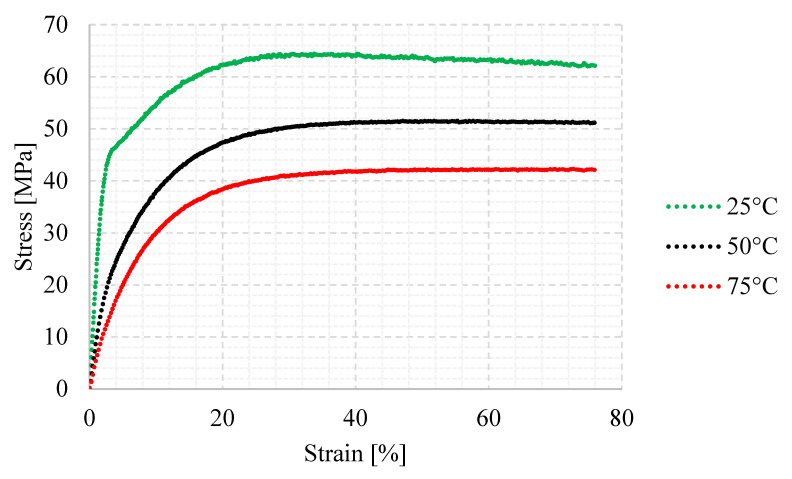
Tensile strength testing curves of 1 BB polyamide 66 specimens for the following ambient temperatures: 25, 50, and 75 °C.

Based on tensile strength testing, strength values were established for each of the tested temperature conditions under which the tests were performed. In Figure 11, the curves for 25, 50, and 75 °C are shown.

The curve representing the tensile test at 25 °C shows a decreasing trend of stress after the maximum stress is achieved. For 50 and 75 °C, the trend is stabilized at a constant level—this might be related to polymer-specific plastic features revealed at elevated temperatures. However, the aim of this testing is to determine the material strength reduction with temperature increase. From a worm gear durability standpoint, stress-over-strain behavior in tensile strength testing is not considered in this article, because deformation of worm gear teeth leads to rigidity loss and meshing errors, resulting in worm gear test interruption.

Tensile strength test results allow us to understand worm gear material strength degradation with temperature increase. This explains the ‘standard test’ poor durability results, as the worm temperature stabilizes at a high level. However, the ‘standard test’ does not reflect regular vehicle steering system utilization, so the test results are not considered valid. ‘Parking test’ results are representative, so they can be used to assess other testing methods’ applicability, like the ‘accelerated test’.

In terms of worm gear durability in a vehicle steering system, the test is understood as finished when the worm gear loses its ability to transmit the assist torque from the assist motor to further steering system components. In this case, the resistive load applied to the rack cannot possibly be overcome. At the test stand, the resultant torque servomotor applies the limit, which corresponds to the EPS system’s assist motor maximum capability. When this value is achieved, the test is stopped.

**Figure 12:**
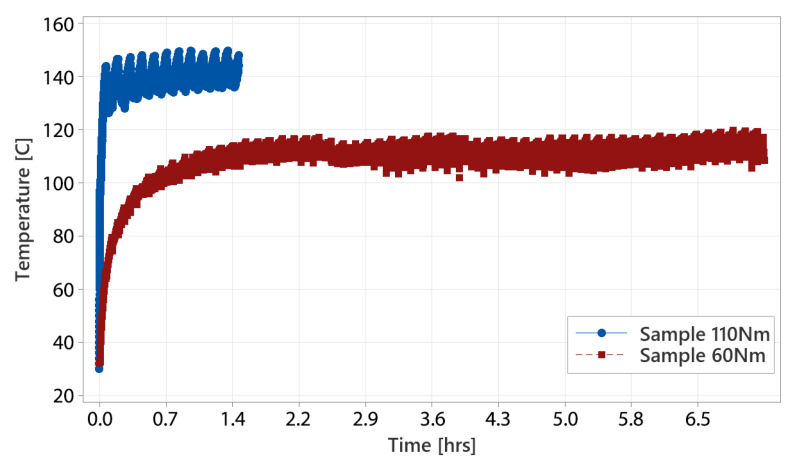
Standard test courses performed at 60 and 110 Nm.

**Figure 13:**
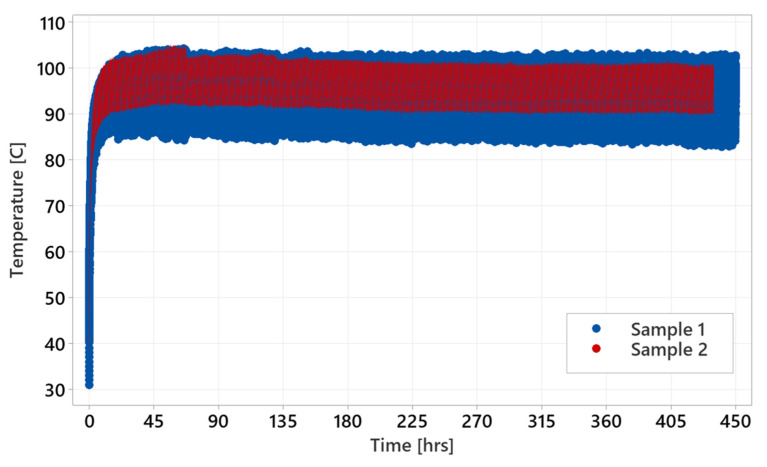
Parking test courses at 110 Nm.

**Figure 14:**
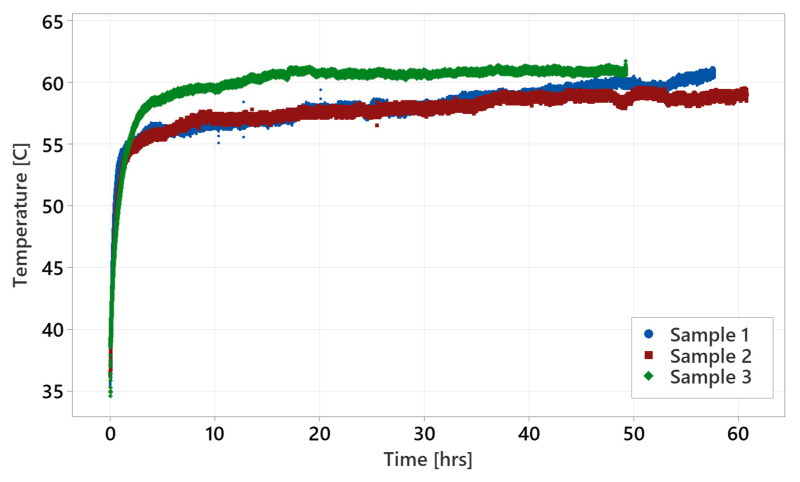
Accelerated test courses at 110 Nm.

The courses for individual test profiles are shown below (Figure 12, Figure 13 and Figure 14). Each shows the temperature record on a timescale. The temperature expresses the difference between specific methods of testing. Figure 12 shows the courses of the ‘standard test’ where the temperature is stabilized around 110 °C for the sample tested at 60 Nm. For the test performed at 110 Nm, worm gear failure occurred before the temperature stabilized, which seems to be too high for constant operation. Figure 13 shows ‘parking test’ courses, where the temperature was stabilized at the beginning and remained stable throughout the entire test. Sample 1 shows slightly larger variation in the recorded temperature than sample 2. However, the temperature average level is around 90 °C for sample 1 and 95 °C for sample 2. The difference does not have a considerable impact on the test result. Figure 14 shows temperature courses for the ‘accelerated test’, where the temperature record has a slight increasing trend after reaching 55–58 °C. Samples 1 and 2 show slightly higher durability performance than sample 3. This might be related to the higher temperature reached during sample 3 testing.

Table 2 shows all of the test results. One cycle means the peak load applied once to the worm gear for its specific position.

**Figure 15:**
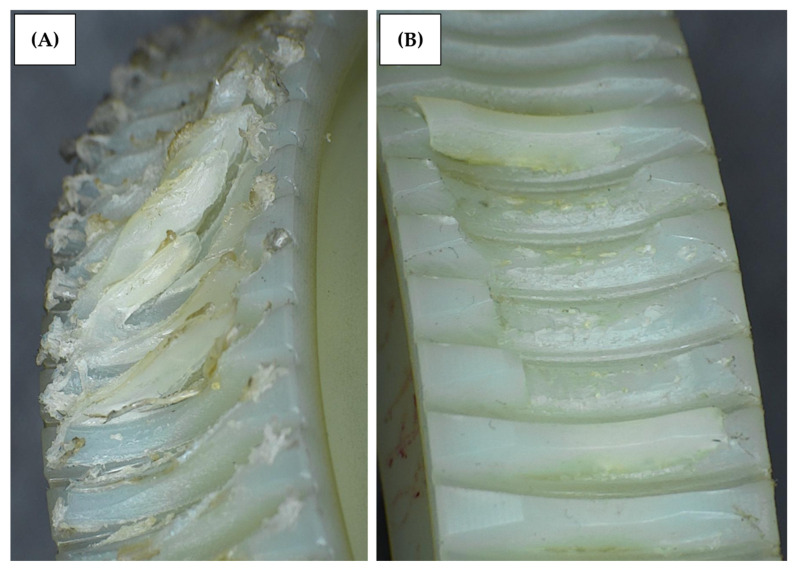
Photo of standard test samples: (**A**) standard test sample at 110 Nm—teeth are deformed and sheared off, and (**B**) parking test sample—teeth fatigue fracture.

**Figure 16:**
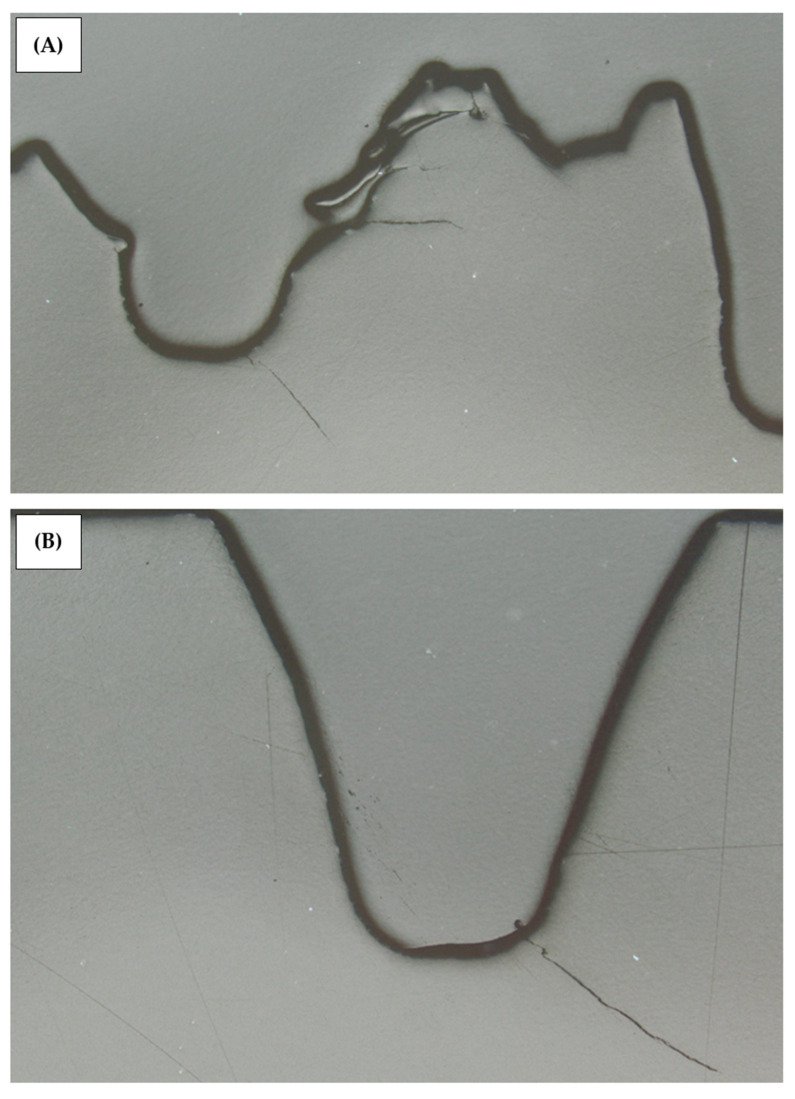
Photo of the parking test sample sectioned: (**A**) fractured tooth and (**B**) fatigue crack near the fractured tooth.

Figure 15 shows an example of a damaged worm gear. Depending on the testing method, the fracture is different. Figure 15A shows the worm gear teeth sheared off, which occurred during a high-temperature run of the standard test. Figure 15B shows the fatigue fracture, which is a common failure for both parking and accelerated tests. Since the standard test does not give a reliable worm gear durability result, the failure was not investigated further. The parking test sample was sectioned in a worm gear center plane and reviewed under a microscope. In Figure 16, the section microscope photos show the worm gear’s fractured area. The worm gear teeth fracture occurred at the area of contact with the worm. Some fatigue cracks in teeth roots were created as well.

## 4. Discussion and Conclusions

Load transferred via worm–worm gear transmission is crucial for its durability performance. Vehicle service life is typically specified by mileage, including specific maneuvers to be performed within it, which are normalized into sequences repeated a certain number of times. The worm gear is a key component that maintains functionality through failure-free operation. Because of this, the appropriate choice of worm gearing design for a specific steering system is critical. Durability testing seems to be the only way to achieve a sufficient confidence level for proper worm gear application. Nevertheless, testing itself has to be performed correctly, reflecting further design utilization, since worm gear transmission behavior during a specific test may reveal different failures that do not occur in real EPS system usage at all. As proven in an experimental way, the temperature achieved during worm gearing operation has a significant impact on its durability. Therefore, teeth stress related to the applied load is not the only factor determining durability. However, the thermal effect is, inter alia, load-driven, which the worm gearing carries. Since different combinations of load and temperature give different results in terms of worm gear teeth damage, specific testing conditions (test profiles) might be separated into several groups. The correlation between different test profiles can be found within a certain group, since the failure mode is the same. Parking and accelerated tests create a group due to the same method of worm gear teeth damage (and the relatively close number of cycles to failure). Standard tests cannot be considered similar and must be grouped separately. The way to reduce excessive thermal effects coming from worm gearing friction heat is, e.g., to test at a lower speed or set pause times between cycles. However, this approach needs to be investigated for its efficiency, because the entire test time seems to be much longer—perhaps just long as an accelerated or parking test.

1. Standard testing applies the load to the tested worm gear in one direction for a long time (many cycles). The sample works in stabilized conditions, which do not occur in any EPS system application.
2. In the parking test, the most stressed worm gear teeth are loaded asymmetrically. The history of loading for certain (most stressed) teeth is complex per one test cycle. Further testing is necessary to understand the damage caused to the worm gear teeth depending on load level, with different combinations of the asymmetry factor. However, asymmetrical loading is reflected in the section photos of the parking test sample—fatigue cracks occur in the direction of high load applied.
3. Worm gear single tooth fracture does not lead to kinematic integrity loss; however, it generates resistance for the worm (assist motor) driving the worm gear, which is indicated by torque peaks during operation. Considering the fact that the EPS system is not fully loaded, the steering system may still work since fractured tooth resistance can be overcome by the assist motor capability. The worm gear becomes useless when all of the teeth supposed to be in temporary contact with the worm are fractured. This situation is inevitable, since a tooth fracture causes overstress to neighboring teeth. Depending on the EPS system working conditions, the assist loss may occur sooner or later.
4. During testing, the resultant torque tracking allows for the detection of potential defects on a worm gear. Applying this logic to the assist motor makes failure detection possible at the vehicle level, increasing product safety, especially important in steer-by-wire systems.
5. Various methods of worm gear durability testing may show some correlation between the gathered results. This might be beneficial in terms of testing time—possibly, the accelerated test could be used as an equivalent manner to define the worm gear durability vehicle system, but it is necessary to understand the impact of the loading asymmetry factor for worm gear durability. Both parking and accelerated tests lead to the same form of worm gear teeth damage, so there is a possibility that a correlation between these methods will be found.
6. The temperature reading on a worm is significantly higher during standard testing than during parking and accelerated testing. This means that reduced durability in standard testing may be related to failures occurring at high temperatures only. This correlates with the reduced PA66 strength in tensile testing at elevated temperatures. In this case, the standard test profile is not applicable since it does not give conclusive results. Perhaps, for further investigation, lower speed and cooling during the test could be considered to keep the worm–worm gear temperature at a lower level that does not lead to premature damage.
7. The method of temperature measurement during testing represents the worm temperature and, somehow, the mesh area. It does not show the effect of heating on the polymer worm gear, whose temperature may be different from the worm. In particular, the cooling time may have an effect on the heat loss of the worm gear, which may significantly impact its durability. The standard test does not contain cooling breaks, which explains the worm temperature growth to a high level and the different form of worm gear damage (where temperature is not controlled). For further testing, extra sensors need to be applied to the worm gear to understand the thermal effect.

## Figures and Tables

**Table 1:** Worm and worm gear basic properties.

Feature	Worm	Worm Gear
Material	42CrMo4 Steel	Polyamide 66
Final manufacturing	Grinding	Hobbing
Outer diameter	16 mm	102 mm
Number of teeth	3	75
Lead angle	19°	4°
Lubrification	Synthetic hydrocarbon grease	

**Table 2:** Test results (number of peak loads until worm gear failure).

Test	Result
Parking test 110 Nm sample 1	29,976
Parking test 110 Nm sample 2	28,900
Standard test 60 Nm	13,120
Standard test 110 Nm	2698
Accelerated test 110 Nm sample 1	21,465
Accelerated test 110 Nm sample 2	22,435
Accelerated test 110 Nm sample 3	18,162

## Data Availability

Restrictions apply to the availability of these data. Data were obtained from Nexteer Automotive Poland and are available at www.nexteer.com with the permission of Nexteer Automotive Poland.
